# Pharmacokinetics of pulmonary indacaterol in rat lung using molecular imprinting solid-phase extraction coupled with RP-UPLC

**DOI:** 10.1038/s41598-024-72822-0

**Published:** 2024-10-04

**Authors:** Mohamed Tarek, Nermine S. Ghoniem, Maha A. Hegazy, Hebatallah A. Wagdy

**Affiliations:** 1 Pharmaceutical Chemistry Department, Faculty of Pharmacy, The British University in Egypt (BUE), Cairo, Egypt; 2 Health Research Center of Excellence, Drug Research and Development Group, Faculty of Pharmacy, The British University in Egypt, Cairo, Egypt; 3 Analytical Chemistry Department, Faculty of Pharmacy, Cairo University, Kasr-El Aini Street, Cairo, 11562 Egypt; 4 Pharmaceutical Chemistry Department, Faculty of Pharmacy, Future University in Egypt, Cairo, 11835 Egypt

**Keywords:** Bioanalytical validation, Indacaterol, Molecularly imprinted polymer, Pulmonary pharmacokinetics, Solid-phase extraction, Ultra-performance liquid chromatography, Analytical chemistry, Bioanalytical chemistry

## Abstract

Indacaterol, a β_2_ agonist prescribed for long-term management of patients with chronic obstructive pulmonary disease and asthma. In this study the first MISPE cartridges was developed using indacaterol as a template for its selective extraction from rat lung tissues, enabling precise pharmacokinetic evaluation at the drug’s site of action. A molecular imprinting polymer was synthesized using indacaterol as a template, methacrylic acid as a functional monomer and ethylene glycol dimethacrylate as a cross-linker with a molar ratio (1: 4: 20). The polymer was characterized by a high binding capacity of 9840 ± 0.86 and high selectivity with an imprinting factor of 4.53 ± 0.12. The synthesized polymer was utilized as a sorbent in solid-phase extraction to purify and extract indacaterol from lung tissue matrix. The optimum molecularly imprinted solid-phase extraction (MISPE) conditions were 20.0 mg of molecular imprinting polymer and non-imprinting polymer, acetonitrile as the loading solvent, acetonitrile: water (20: 80; *by volume*) as the washing solvent, and methanol: acetic acid (90: 10; *by volume*) as the eluting solvent. A pharmacokinetic study was performed for indacaterol in rat lungs using the synthesized and optimized MISPE cartridge as a tool for sample purification. These parameters were determined in the lung tissues of rats emphasizing the local exposure of indacaterol to its target organ. The C_max_ and T_max_ were 51.020 ± 2.810 µg mL^− 1^ and 0.083 ± 0.001 h, respectively. The AUC _0−24_ and AUC_0 − inf_ were 175.920 ± 1.053 and 542.000 ± 5.245 µg h mL^− 1^, respectively. The elimination rate constant was 0.014 ± 0.00012 h^− 1^ and the half-life time was 48.510 ± 0.012 h. This study successfully developed and optimized MISPE cartridges using indacaterol as a template, enabling precise pharmacokinetic evaluation in rat lung tissues. The cartridges demonstrated high binding capacity and selectivity, providing crucial insights into the local exposure of indacaterol at its site of action.

**Supplementary Information:**

The online version contains supplementary material available at 10.1038/s41598-024-72822-0.

## Introduction

**Fig. 1:**
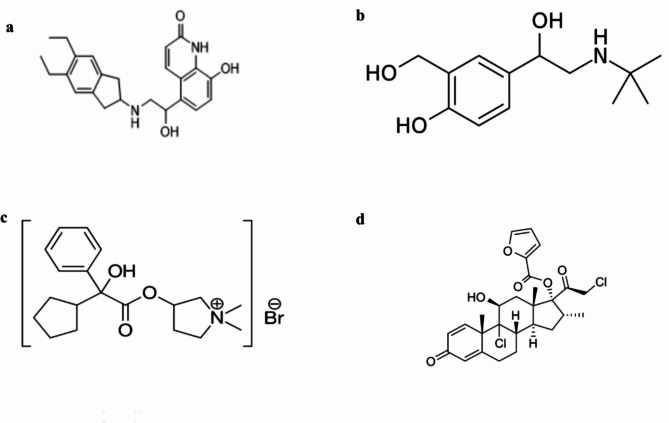
Chemical structure of (**a**) indacaterol, (**b**) salbutamol, (**c**) glycopyrronium bromide and (**d**) mometasone furoate.

Indacaterol 5-[(1R)-2-(5,6-diethyl-indan-2-ylamino)-1-hydroxy-ethyl]−8-hydroxy-1H-quinolin-2-one), Fig. 1a^1^, is a β_2_ agonist inhaler that is designed to provide long-term management of asthma and chronic obstructive pulmonary disease (COPD)^2,3^. The stimulation of intracellular adenyl cyclase, an enzyme responsible for converting adenosine triphosphate (ATP) into cyclic 3’, 5’ adenosine monophosphate (AMP), is thought to be the cause of indacaterol’s physiological effects^4^. The bronchial smooth muscle relaxes because of this rise in cyclic AMP levels. Indacaterol is taken once daily in doses ranging from 75 to 600 µg. This decrease the frequency of administration and increase the patient’s compliance which distinguishes it from other long-acting inhaled β_2_ agonists like formoterol and salmeterol^5,6^.

Indacaterol is given via inhalation device to maximize bronchodilation activity and reduce systemic exposure. As a result, rather than relying on the drug’s concentration in the bloodstream, which could not be directly related to the desired therapeutic impact, the efficiency of the treatment depends on the drug’s concentration in the local tissue^7^. Due to its high lipophilicity, indacaterol has a strong affinity for lipid membranes and gets partitioned into the receptor microenvironment. Additionally, indacaterol has a two-fold higher binding affinity for raft micro-domains, which are small invaginations (50/100 nm) of the plasma membrane in the smooth muscle of the airway, compared to other β_2_ agonists. This feature could contribute to the rapid onset of action and sustained presence of indacaterol at the targeted action site^8^. After being inhaled, indacaterol enters the bloodstream within 5 min and achieves its highest concentration approximately 15 min later, particularly at very low doses, before undergoing subsequent elimination. The absolute bioavailability of indacaterol, the ratio between drug concentration in lung and the intravenous concentration, is 45% and the fraction of systemically available indacaterol due to pulmonary absorption is 74% (F_lung_). Accordingly, the percentage of indacaterol dose that is absorbed and deposited in the lung, estimated as absolute bioavailability multiplied by F_lung_ is 34%, providing evidence of effective lung delivery of inhaled indacaterol^7^. The steady state of indacaterol is achieved after 12 days of once-daily inhalation. Consequently, assessing the levels of indacaterol at the site of action is important for investigating the drug’s dosage availability in the lungs following oral inhalation.

There are various methods for drug extraction and purification from biological matrices, but for complex tissues like lung, solid-phase extraction (SPE) is preferred due to its efficiency and low solvent use^9^. Traditional SPE can suffer from non-specific interactions, leading to co-extraction of contaminants. Molecularly imprinted polymers (MIP) offer a solution with high selectivity for the target analyte. MIP creates selective binding sites by arranging monomers around a template molecule, followed by polymerization and template removal, resulting in cavities that specifically recognize the target analyte^10^. MIP exhibits high selectivity, stability, and reusability and can be stored for extended periods. As a result, they have found applications in a variety of fields, such as separation and purification of compounds^11–13^, drug delivery^14^, and biosensors^15–17^.

Different analytical techniques have been described in the literature for the analysis of indacaterol using spectrophotometry^18,19^ and Spectrofluorimetry^19^. High-performance liquid chromatography (HPLC) coupled with UV detector^20,21^, mass spectrometer detector^22–24^, as well as stability-indicating assay methods using HPLC^25,26^. SPE using conventional sorbents has been employed to purify indacaterol from different matrices, including plasma^24,27,28^ and urine^29,30^ followed by analysis using liquid chromatography mass spectrometric methods. For instance, there is no method reported for the analysis of indacaterol using the MIP technique. Also, different pharmacokinetic studies were carried out for indacaterol in plasma^7,23,24,27^ but, no pharmacokinetic study was developed for indacaterol in its site of action, which is the lung tissues.

Consequently, the main purpose of this investigation is to develop the first molecular imprinting solid-phase extraction (MISPE) cartridges contained a polymer using indacaterol as a template for polymer preparation and employ these cartridges for the selective extraction of indacaterol from pulmonary tissues of rats, marking the first utilization of such cartridges for this purpose. This technique will enable the pharmacokinetic evaluation and the determination of indacaterol deposition at its site of action within the lungs. Furthermore, this investigation seeks to establish the pharmacokinetic parameters of indacaterol within pulmonary tissues, including C_max_, T_max_, AUC _0−24_, AUC _0−inf_, elimination rate constant, and half-life time. To accomplish these objectives, a bioanalytically validated ultra-performance liquid chromatographic method was developed for the analysis of indacaterol in the obtained samples.

## Materials and methods

### Chemicals and reagents

Indacaterol standard was purchased by Sigma-Aldrich (Steinheim, Germany), glycopyrronium was provided by Novartis Company (Cairo, Egypt), mometasone was provided by EVA pharmaceuticals (Cairo, Egypt) and salbutamol was provided by National Organization for Drug Control and Research (NODCAR, Cairo, Egypt) they were certified to contain 99.92 ± 0.04, 99.96 ± 0.01, 99.93 ± 0.02 and 99.98 ± 0.05% drug, respectively. The pharmaceutical preparation, Onbrez Breezhaler capsules contain 150 µg indacaterol manufactured by Novartis Company, batch number BDKH6 was purchased from the Egyptian market.

Methacrylic acid (MAA), ethylene glycol dimethacrylate (EDMA), 4-vinyl pyridine (4-VP), 2, 2- azobisisobutyronitrile (AIBN) were purchased from Sigma-Aldrich (Steinheim, Germany). HPLC grade methanol, acetonitrile, dimethyl sulfoxide (DMSO), ethanol, dichloromethane (DCM) and chloroform and analytical grade ortho-phosphoric acid, monobasic sodium phosphate, dibasic sodium phosphate, methanol, ammonia, and glacial acetic acid were provided by Fisher Scientific (Loughborough, Leicestershire, UK). Phosphate buffer saline (PBS) was provided by Sigma-Aldrich (Steinheim, Germany).

### Animals

Twenty-four adult and male Wistar rats, 12–14 weeks of age with weight of 200–220 g, bred in the animal facility of the Faculty of Pharmacy, The British University in Egypt (BUE; Cairo, Egypt) were used. All animals were housed in plastic cages (3 rats/cage), kept at a temperature of 23 ± 2^º^C, a humidity of 40–60%, 12/12 h light/dark cycle (light on at 6 a.m.), food and water were given ad libitum. Animals handling and experiment were conducted and approved according to the guidelines of the Research Ethics Committee of the Faculty of Pharmacy, the British University in Egypt (Approval No. CH-2306). The ethics committee was registered and recognized by the Egyptian Network of Research Ethics Committees (ENREC). The study is reported in accordance with ARRIVE (Animal Research: Reporting of In Vivo Experiments) guideline.

### Instruments

The used UPLC system was Thermo Fisher UHPLC Dionex Ultimate 3000 (Germering, Germany) using software Chromeleon version 6.8 (Germering, Germany). The chromatographic separation was conducted using an Hypersil gold C18 column, 2.2 μm (2.1 × 100 mm). pH was adjusted using a pH-meter (Jenway-pH-meter-3310, Dunmow, Essex, UK). De-ionized water was generated by water purification system (Thermo scientific, Barnstead Smart-2 Pure 3-UV, Hungary).

A digital shaking water bath (Wisd, WSB 18, Berlin, Germany) was used for polymer preparation. Freeze centrifuge (Sigma, 2–16, KL, Germany) was used for samples centrifugation. The polymer was characterized by Fourier transform infrared (FTIR), Shimadzu IR 435 spectrophotometer (Kyoto, Japan) and scanning electron microscope (SEM) ThermoFisher (USA), Quattro-S-Felid Emission Gun, Environmental SEM (FEG-ESEM). Stainless steel sieves of particle size 90 and 75 μm (Egyptian industry, Cairo, Egypt) were used to adjust polymer particle size.

The SPE manifold is connected to a Vacuum Pump, 220-V/240-V, 50 Hz and an empty cartridges (3 mL/9 mm diameter) containing polyethylene frits were supplied from (Waters/Milford, USA).

Lung tissues were homogenized using (Silent Crusher Homogenizer, Heidolph Instruments, Germany). Centrifuged using (Centurion, K241R, UK). Evaporation of samples was achieved using a rotatory concentrator containing a vacuum pump (DVP/TYRO/12, Germany), solvent trap (Christ-CT-02-50, Germany) and rotor (Christ-RVC-2-18, CDplus, Germany). Vortex of samples was performed using (Velp Scientifica, Europe) and sonicated using an ultra-sonicator (Elmasonic S 60 (H), Germany). Used euthanasia was performed using carbon dioxide using (CS-EUTH2A-2, Conduct science, USA).

### Samples preparation

#### Standard solutions preparation

The stock standard solutions of indacaterol, salbutamol, Fig. 1b, glycopyrronium Fig. 1c, and mometasone Fig. 1d were prepared by accurately weighing 10.00 mg from each of the standards separately in 10-mL volumetric flask and diluted using methanol for indacaterol, de-ionized water for glycopyrronium, and acetonitrile for salbutamol and mometasone to prepare 1.0 mg mL^− 1^ for each drug. The stock standard solutions were kept at 4 °C.

#### Preparation of quality control samples and lung tissue samples

LLOQ (lower limit of quantification, 0.10 µg mL^− 1^) and quality control samples (QCs): QCL (low quality control, 0.30 µg mL^− 1^), QCM (medium quality control, 35.0 µg mL^− 1^) and QCH (high quality control, 70.0 µg mL^− 1^) were prepared by diluting 1.0, 3.0, 350.0 and 700.0 µL from indacaterol standard stock solution with acetonitrile into separate 10.0 mL volumetric flasks.

Regarding the preparation of lung tissue samples, three non-medicated male rats were sacrificed by decapitation and their whole lungs were harvested. The lungs were cut into small slices and mixed with 2.0 mL of PBS, homogenized in an ice jacket for 10.0 min. Subsequently, 1800.0 µL of the homogenate was collected and spiked with 200.0 µL of indacaterol solution with concentrations of 1.0, 3.0, 350.0, and 700.0 µg mL^− 1^ separately, to achieve the concentration of the LLOQ and QCs per 1.0 mL of the lung homogenate. Samples were centrifuged at 3000 rpm for 10 min and 1.0 mL of the supernatant was passed through the MISPE cartridge according to the extraction protocol outlined in section "Preparation and optimization of MISPE". Finally, 10.0 µL of salbutamol stock standard solution was added to the eluent as IS, and the samples were injected into the UPLC for analysis.

### Analytical procedures

#### Chromatographic conditions

The method used for indacaterol analysis was established and validated according to previously published research by our group^31^, using Hypersil gold C18 column, 2.2 μm (2.1 × 100 mm) as a stationary phase, methanol and acidified water adjusted to pH (5.0) using 1.0% ortho-phosphoric acid (50: 50, *by volume*) as a mobile phase. The column oven was adjusted at 35°C. Injection volume was 10.0 µL, flow rate was 0.35 mL min^− 1^ and the UV detection was at 220 nm.

#### Bioanalytical method validation

The previously mentioned method was subjected to bioanalytical validation according to the FDA guidelines^32^ for the analysis of indacaterol in the lung matrix.

For evaluating the linearity, a calibration graph was constructed using indacaterol with concentrations of 0.10, 0.50, 1.0, 5.0, 10.0, 20.0, 50.0, 60.0, 80.0, 100.0 µg mL^− 1^. The calibration graph was constructed using the analyte to IS peak area ratios against the analyte concentration. The acceptance criteria for a calibration graph were evaluated by the regression coefficient (R²).

The method selectivity was evaluated by injecting six blank (lung sample without IS and the drug), zero (lung sample processed with IS only) and non-zero lung tissue samples separately in different days to check for any interfering substance from the lung matrix with indacaterol and IS.

Intra-day accuracy and precision were evaluated by injecting LLOQ and QCs six times in the same day. While, in inter-day accuracy and precision samples were injected six times in six different days.

% recovery (% R) was used to evaluate the accuracy and it was calculated by Eq. (1)

1 $$\frac{{Mean\;found~\;concentration}}{{Prepared\;~concentration}} \times 100$$

The acceptable range for % R and % relative standard deviation (% RSD) is ± 15.0%.

Samples stabilities were assessed by injecting LLOQ and QCs six times and compared to freshly prepared samples using the following stability conditions:

##### Short-term stability

Samples were left at room temperature for a time exceeding that required for sample preparation (2 h).

##### Auto-sampler stability

Samples were left in autosampler (30°C) for 6 h; expected time of samples storage in autosampler before injection. Then, samples were injected.

##### Freeze and thaw stability

Samples were subjected to three cycles of freeze and thaw each of 24 h and the samples were kept under the same conditions as during the analysis.

##### Long-term stability

Samples were stored at -80^º^C over a period exceeding that required for the whole experiment (3 weeks).

The results of all stability conditions were evaluated using Eq. (2). The deviation should not exceed ± 15.0%.

2 $$\frac{{\% ~\;R\;of\;stored\;sample - \% ~R\;~of\;~the\;~freshly\;~prepared~\;sample}}{{\% ~R~\;of~\;the\;~freshly~\;prepared~\;sample}} \times 100$$

The efficiency of extraction of indacaterol from the lung tissues was evaluated by comparing the concentration of LLOQ and QCs spiked to lung tissues before the extraction procedures mentioned under section "Preparation of quality control samples and lung tissue samples" to the concentration of LLOQ and QCs spiked to lung tissue samples after extraction procedures mentioned under section "Preparation of quality control samples and lung tissue samples" as in Eq. (3).

3 $$\frac{{peak\;~area\;~of\;~the~\;QC~\;samples\;~spiked\;~to~\;lung~\;samples~\;before~\;extraction}}{{peak\;~area~\;of\;~the~\;QC~\;samples~\;spiked\;~to~\;lung~\;samples~\;after~\;extraction}} \times 100$$

### MIP synthesis and characterization

A thermal free-radical bulk polymerization method was used for the MIP synthesis. An initial step involved dissolving 1.0 mmol of indacaterol (template) in 10.0 mL of porogen, which consisted of DMSO and acetonitrile in a (1: 1; *by volume*) ratio. The monomer (MAA) or (4-VP) was then added to the solution to form pre-polymerization complex then, the cross-linker (EDMA) was subsequently added in a molar ratio of template: monomer: cross-linker (1: 4: 20). The mixture was shaken for 5 min, and then 0.10 g of the initiator (AIBN) was added to initiate the polymerization. The mixture was purged with nitrogen for 10 min before being added to a paraffin oil bath adjusted at 60°C for 24 h. The resulting polymer was ground and sieved to achieve particles of diameter ranging from 75 to 90 μm. The polymer was washed using a mixture of methanol: acetic acid (90: 10; *by volume*) for several cycles each of 2 h to extract the template molecules and UPLC was used to monitor the template concentration in the washing solution. This extraction procedure was repeated till the absence of template molecules in the washing solution. The polymer was then washed with methanol and allowed to dry at room temperature. A non-imprinted polymer (NIP), which served as a blank, was synthesized in the same manner as MIP without the presence of template molecule^33^.

#### Batch rebinding procedures

A weight of 20 mg of MAA and 4-VP based MIP and their corresponding NIP were separately weighed into Eppendorf tubes. The tubes were then incubated with 2 mL of a 60.0 µg mL^− 1^ indacaterol standard solution, which was prepared by diluting 600.0 µL of 1.0 mg mL^− 1^ indacaterol stock solution in 10.0 mL of acetonitrile. The Eppendorf tubes were shaken at 250 rpm for 2 h at room temperature. Subsequently, the polymer was filtered using a 0.22 μm nylon syringe filter, and the concentration of indacaterol in the filtered solution was quantified using UPLC. The binding capacity (Q, µg g^− 1^) for MIP and NIP were calculated according to Eq. (4)^34^:

4 $$Q=\frac{{\left( {{C_i} - {C_f}} \right) \times V}}{M}$$

where, Q is the maximum indacaterol amount that can be bound to the weighed polymer, C_i_ is the initial indacaterol concentration (µg mL^− 1^), C_f_ is the free indacaterol concentration after binding to the polymer (µg mL^− 1^), V is the volume of indacaterol solution added to the polymer (mL) and M is the mass of polymer used (g). The binding capacity of MIP (Q_MIP_) was compared with that of NIP (Q_NIP_) to determine the polymer selectivity in term of imprinting factor (IF) according to Eq. (5)^34^:

5 $$IF={Q_{MIP}}/{Q_{NIP}}$$

#### Effect of solvent on rebinding

Various solvents including methanol, acetonitrile, water, ethanol, DMSO, DCM, and chloroform were used to prepare a 60.0 µg mL^− 1^ indacaterol solution. This was achieved by diluting 600.0 µL of a 1.0 mg mL^− 1^ indacaterol stock solution in 10.0 mL of each solvent separately. Subsequently, 2.0 mL of each prepared solution was added separately to 20.0 mg of MIP and NIP, following the same procedures as batch rebinding (2.6.1). The outcomes of each solvent effect were evaluated using the Q and IF parameters.

#### Binding kinetics

It was performed by transferring 20.0 mg of MIP and NIP separately into Eppendorf tubes. Then, 2.0 mL of 60.0 µg mL^− 1^ indacaterol prepared in the optimal solvent was added, shaken at 250 rpm for different time intervals 5, 15, 30, 60, 120, 240, 360, 480, 1080, 1200 and 1440 min. After each time, MIP and its corresponding NIP were filtered by 0.22 μm nylon syringe filter, the concentration of indacaterol in the filtrated solution was measured using UPLC. The results were evaluated using Q and IF.

#### Binding capacity and affinity

Aliquots of 20.0 mg of MIP and NIP were incubated with 2.0 mL of indacaterol solution prepared using the optimal solvent with different initial concentrations which were 0.10, 0.50, 1.0, 5.0, 10.0, 20.0, 50.0, 60.0, 80.0 and 100.0 µg mL^− 1^ for 2 h, using the same procedures of the batch rebinding studies (2.6.1). The quantification was done using UPLC. Hence, the corresponding Q and IF were determined.

Binding isotherm was determined by plotting concentrations of indacaterol (µg mL^− 1^) versus the binding capacities (Q, µg g^− 1^) for MIP and NIP. While the characterization of the binding sites was studied using Scatchard plot according to Eq. (6).

6 $$Q/{C_f}={Q_{max}} - Q/{K_D}$$

where Q is the binding capacity of the polymer (µg g^− 1^), C_f_ is the free concentration of indacaterol (µg mL^− 1^), Q_max_ is the maximum number of binding sites in (µg g^− 1^), K_D_ is the equilibrium dissociation constant (µg).

The binding model of indacaterol toward the polymer was determined using Langmuir isotherm model Eq. (7) and Freundlich isotherm model Eq. (8).

7 $${C_f}/Q=1/{Q_{max~}}{K_L}+\frac{{{C_f}}}{{{Q_{max}}}}$$

8 $$~LnQ=Ln{C_f}/n+Ln{K_F}$$

where K_L_ Langmuir constant in (mL µg ^− 1^), K_F_ is Freundlich constant in (µg^1 − 1/n^ mL^1/n^ g^− 1^) and n is adsorption affinity of the polymer towards indacaterol^35^.

#### Polymer selectivity

To investigate the selectivity of the polymer towards indacaterol, a competitive binding study was conducted with glycopyrronium and mometasone. A mixture of the three drugs with a concentration of 40.0 µg mL^− 1^ for each drug was prepared by diluting 400.0 µL from the stock solution of each drug in a 10.0 mL volumetric flask using acetonitrile. Next, 2.0 mL of the mixture was added to 20.0 mg of the prepared MIP and NIP, following the same procedures as described in the batch rebinding section "Batch rebinding procedures". The concentrations of the three drugs were determined using the same UPLC method, and Q and IF were calculated for each drug.

A similar approach was taken to investigate the selectivity of the polymer towards indacaterol in the presence of salbutamol. A mixture of the two drugs with a concentration of 40.0 µg mL^− 1^ for each drug was prepared by diluting 400.0 µL from the stock solution of each drug in a 10.0 mL volumetric flask using acetonitrile. Afterward, 2.0 mL of the mixture was added to 20.0 mg of indacaterol polymer MIP and NIP, following the same procedures as described in the batch rebinding section "Batch rebinding procedures". The concentrations of the two drugs were determined using the same UPLC method, and Q and IF were calculated for each drug.

#### Polymer characterization

To characterize indacaterol and MIP before and after indacaterol extraction, as well as its corresponding NIP, FTIR spectra were recorded in the range of 5000 to 400 cm^− 1^. Also, the surface morphology of the MIP and NIP were analyzed using SEM.

### Preparation and optimization of MISPE

MIP was used as a selective sorbent for the extraction of indacaterol. It was packed in an empty 3 mL and 9 mm diameter cartridges with polyethylene frits to secure the MIP in place. In all MISPE procedures NIP packed the same way as MIP to be used as a blank and the flow rate was 0.5 mL min^− 1^.

#### Polymer weight

Various weights of both MIP and NIP included 5.0, 10.0, 15.0, 20.0, 25.0 and 30.0 mg were tested. The MISPE cartridge was conditioned with 1.0 mL of acetonitrile. It was then loaded with 1.0 mL of 60.0 µg mL^− 1^ indacaterol standard in acetonitrile, and the concentration of indacaterol was measured using UPLC after the loading step. The percentage of the loaded drug to both MIP and NIP were calculated.

#### Cartridge capacity

It was evaluated by loading 1.0 mL of indacaterol solution with different concentrations of 0.10, 0.50, 1.0, 5.0, 10.0, 20.0, 50.0, 60.0, 80.0 and 100.0 µg mL^− 1^ prepared in acetonitrile, into both the MIP and NIP using the same procedures described in section "Polymer weight". The percentage of the loaded drug was determined for each concentration.

#### Loading solvent

Indacaterol solutions with a concentration of 60.0 µg mL^− 1^ were prepared by diluting the indacaterol stock solution in various solvents, including methanol, acetonitrile, and phosphate buffer at pH values of 3.0, 5.0, and 8.0. Using the same procedures described in section "Polymer weight", one milliliter of each solution was loaded separately onto both the MIP and NIP. The percentage of the loaded drug was determined for each solution.

#### Washing solvent

MIP and NIP cartridges were loaded with 1.0 mL of an indacaterol solution with a concentration of 60.0 µg mL^− 1^ prepared in acetonitrile. The cartridges were washed with various solvents, including water, acetonitrile: water (80: 20, 20: 80 and 50: 50; *by volume*) and methanol: water (80: 20, 20: 80 and 50: 50; *by volume*). The concentrations of indacaterol in the washing eluate were determined using UPLC and the percentage of the washed drug was calculated.

#### Eluting solvent

Several eluting solvents were evaluated for their ability to elute indacaterol from MISPE cartridge. These solvents were mixtures of methanol and acetic acid at ratios of (70: 30, 80: 20, and 90: 10; *by volume*), as well as acetonitrile and acetic acid at a ratio of (90: 10; *by volume*) and methanol and ammonia at a ratio of (90: 10; *by volume*). The concentration of indacaterol in the eluent was determined using UPLC and the % R was determined regarding the initial concentration of indacaterol used in the loading step.

#### Cartridge selectivity

The selectivity of the cartridge was investigated in a similar manner as mentioned in the "Polymer selectivity" section. In this study, 1.0 mL from a mixture of indacaterol, glycopyrronium, and mometasone was loaded to MISPE cartridge, and the percentage of each drug loaded, washed, and eluted were determined. The same procedure was repeated using 1.0 mL mixture of indacaterol and structurally related salbutamol. The percentages of each drug loaded, washed, and eluted were also determined.

#### Cartridge reusability

The maximum number of times that MISPE cartridges could be utilized was determined by calculating the % R after the elution step for indacaterol standard and indacaterol spiked into lung tissues during the bioanalytical validation.

### Pharmacokinetic study of indacaterol in rat lung tissues

In this study, male Wistar rats (*n* = 24) were used to investigate the lung deposition of indacaterol and determine the lung concentration-time curve and pharmacokinetic parameters including C_max_ and T_max_ (the maximum concentration of indacaterol in lung tissues and the time at which indacaterol reached its maximum concentration in the lung, respectively), AUC _0−t_, AUC _0−inf_ (area under the lung concentration-time curve from time zero to 24 h, and from zero to infinity, respectively) t_1/2_ (half-life time) and elimination rate constant. AUC was calculated using the trapezoidal rule where, AUC _0−t_ was determined by summing the areas of consecutive trapezoids formed by plotting drug concentration versus time^36^. The elimination rate constant was estimated from the least-squares regression slope of the terminal lung concentrations by multiplying the negative slope by 2.303^36^. The AUC _0−inf_ was calculated using Eq. (9)^36^:

9 $$AU{C_{0 - inf}}=AU{C_{0 - t}}+{C_t}/Elimination~\;rate~\;constant$$

where, C_t_ is the last lung concentration measured. The t_1/2_ was calculated using Eq. (10)^36^:

10 $${t_{1/2}}=0.693/Elimination\;~rate\;~constant$$

The rats were divided into eight sampling time points (*n* = 3/sampling time point) representing different time intervals (0.0, 5.0, 15.0, 30.0, 60.0, 180.0, 360.0 and 1440.0 min) for the pharmacokinetic study. Except for the zero-time sampling point; where rats were not administered the drug and were used for bioanalytical validation; each rat in each sampling time point was administered one inhalation capsule containing 150 µg of indacaterol using a powder administration device for animals (Aptar Pharma, Le Prieure, Le Neubourg, France). The powder administration device was designed to deliver a uniform dose of dry powder inhalers into the lungs of rats.

At each time interval, three rats were subjected to euthanasia in the euthanasia chamber using carbon dioxide gas inhalation, after being unconscious, the rats were subjected to decapitation and their lungs were separated and cut into small slices to facilitate homogenization. Then, the lung tissues were homogenized for 10 min using a homogenizer placed in an ice jacket with 2.0 mL of PBS. The homogenized samples were then centrifuged, and the supernatant was separated and loaded into MISPE cartridge following the optimized MISPE steps. The drug was eluted in 1.0 mL of the eluent. Then, 10.0 µL of salbutamol stock solution was added to the eluent as an IS, and the eluent was then injected into UPLC. The same procedure was repeated at each time interval. Dead animal bodies were kept in biohazard bags in -20^º^C freezer and finally incinerated by specialized waste disposal companies.

## Results and discussion

In this study, a polymer was synthesized with indacaterol as a template. This polymer was then used as a sorbent for the development of MISPE cartridge. The primary aim was to utilize this approach for the selective extraction and purification of indacaterol from lung tissues of rats. By achieving this, the study enabled the first pharmacokinetic assessment of indacaterol at its site of action. Additionally, it facilitated the calculation of indacaterol’s lung deposition, as well as the determination of its pharmacokinetic parameters and understanding indacaterol’s behavior in the lungs.

### Analytical procedures

The UPLC method used in this study was previously developed and validated by our research group^31^. The used stationary phase was Hypersil gold C18 column, 2.2 μm (2.1 × 100 mm). The mobile phase was methanol and acidified water adjusted to pH (5.0) using 1.0% ortho-phosphoric acid (50: 50, *by volume*). The column oven was adjusted at 35°C. Injection volume was 10.0 µL, flow rate was 0.35 mL min^− 1^ and the UV detection was at 220 nm.

### Bioanalytical validation

The developed method was bioanalytically validated using the prepared LLOQ and QCs as mentioned under section "Preparation of quality control samples and lung tissue samples". Where, The QCL concentration was set to three times the LLOQ, while the QCH concentration was established at 70% of the highest concentration in the calibration curve. Finally, the QCM concentration was 50% of the QCH. The developed method was linear for indacaterol concentrations ranging from 0.10 to 100.0 µg mL^− 1^ using salbutamol as IS as shown in the calibration curve Fig. S1. The R^2^ was 0.9964 and LLOQ concentration was 0.10 µg mL^− 1^ with accuracy of 112.00% and the % RSD was 5.02%.

**Fig. 2:**
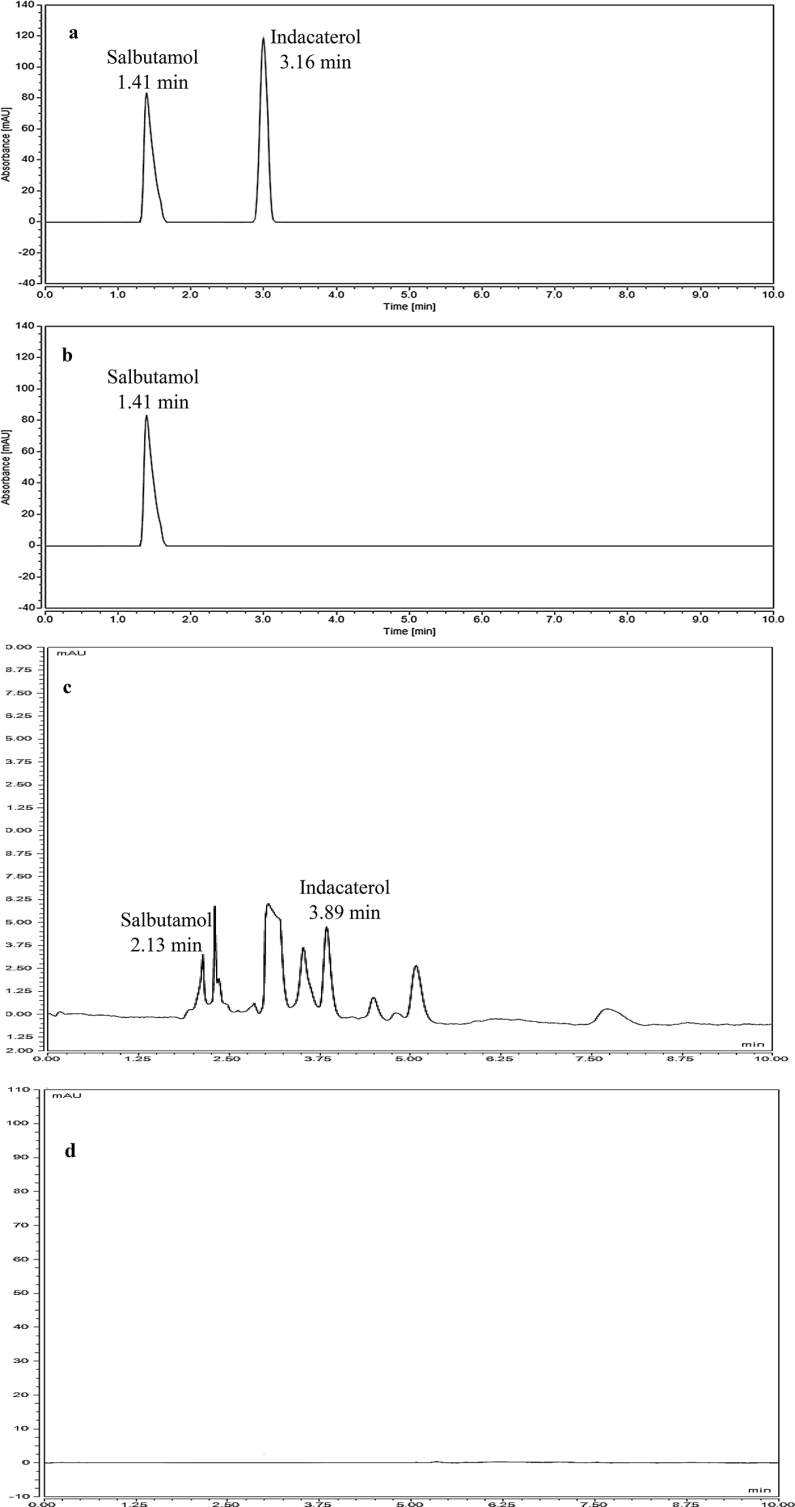
UPLC chromatograms of (**a**) 60.0 µg mL^− 1^ indacaterol and 10.0 µg mL^− 1^ salbutamol as IS using MISPE, (**b**) zero sample spiked with 10.0 µg mL^− 1^ salbutamol as IS using MISPE, (**c**) lung tissue sample spiked with 60.0 µg mL^− 1^ indacaterol and 10.0 µg mL^− 1^ salbutamol as IS using protein precipitation technique and (**d**) blank lung tissue sample using MISPE.

The method was selective as it was able to separate indacaterol in the presence of salbutamol as IS without the interference as represented in Fig. 2a. Also, zero sample chromatogram; blank lung matrix spiked with IS; Fig. 2b showed no interference from any substance. Both chromatograms were developed using MISPE technique.

The method was accurate as the % R was in the acceptable range between 101.36 and 112.00% for the intra-day accuracy and between 102.17 and 113.05% for the inter-day accuracy. The method showed high precision evidenced by % RSD between 1.95 and 4.34% and 2.19–5.02% for intra-day and inter-day precisions, respectively as represented in Table S1.

Samples showed good stability during short-term stability, auto-sampler stability, freeze-thaw stability, and long-term stability as shown in Table S2.

The extraction protocol of indacaterol from lung tissue samples showed high efficiency as represented in Table S3. Where % R of indacaterol after extraction from lung tissues using MISPE technique was high compared to that of the pure standard analyte.

### Synthesis and characterization of MIP

Molecular imprinting is a widely used technique for synthesizing artificial receptors, and bulk polymerization is a cost-effective and straightforward method for generating MIP without requiring complex instrumentation. Prior to polymerization, all reaction components were dissolved in a solvent, which is referred to as a porogen. The solvent impacts the morphology of the final MIP, the stability of the pre-polymerization complex, the size and the availability of the pores and its distribution. In this study, the porogenic property of DMSO was investigated for the synthesis of MIP. Although DMSO was capable of dissolving indacaterol, the polymer obtained was sticky and could not be sieved due to the high boiling point of the solvent, which made it difficult to evaporate the solvent completely. As a result, traces of DMSO remained after polymer formation. While the acetonitrile and chloroform were found to be unsuitable due to indacaterol’s insolubility. Also, methanol was not chosen as a porogen due to its highly polar nature, which disrupted the hydrogen bond interaction between indacaterol and the functional monomer, resulting in an unstable pre-polymerization complex. Therefore, the best porogen was a non-polar solvent and able to dissolve indacaterol which was a 10.0 mL mixture of acetonitrile and DMSO (1: 1; *by volume*). Additionally, the polymer was purged with nitrogen before the thermal polymerization to remove oxygen, which can damage the binding sites. After indacaterol extraction from MIP using methanol: acetic acid (90: 10; *by volume*), the prepared polymer was washed twice with 50.0 mL of methanol to remove any excess acetic acid that could affect the binding sites.

#### Batch rebinding procedures

A batch rebinding study was conducted to evaluate the imprinting behavior, binding capacity, and selectivity of the synthesized polymers. Functional monomer is responsible for the reaction with the template to form a pre-polymerization complex. Accordingly, MAA, an acidic monomer, and 4-VP, a basic monomer, were used in a molar ratio of (1: 4: 20) for polymer preparation. This choice was based on the presence of basic amino groups and acidic hydroxyl groups in indacaterol, which can form hydrogen bonds with MAA and 4-VP. Additionally, EDMA was used as the cross-linker to maintain the steric arrangement of the template and monomers and stabilize the formation of binding sites. The ratio of monomer and cross-linker was kept higher than that of the template to shift the reaction towards polymer formation based on Le Châtelier principle. This ratio was reported as the most used in literature^37^. Increasing the ratio of monomers led to the formation of intra-molecular hydrogen bonds between monomer molecules, resulting in the formation of non-specific binding sites. Similarly, an increase in the ratio of the cross-linker led to the formation of a rigid polymer where binding sites were inaccessible for the template.

As shown in Tables 1 and 4-VP-based polymer exhibited lower Q and IF compared to MAA-based polymer, indicating a stronger interaction between indacaterol hydroxyl group and MAA carboxylic group. The results also demonstrated that MAA-based polymer exhibited high binding capacity and selectivity towards indacaterol, with Q and IF values of 9840 ± 0.86 and 4.53 ± 0.12, respectively. This suggests that the synthesized MIP has the potential to be used as a selective and efficient tool for indacaterol identification.

**Table 1:** Binding capacity and imprinting factor of the prepared polymers.

Monomer	Template: monomer: cross-linker	Q_MIP_ (µg g^− 1^)^*^	Q_NIP_ (µg g^− 1^)^*^	IF^*^
MAA	1: 4: 20	9840 ± 0.86	2172 ± 0.74	4.53 ± 0.12
4-VP		3668 ± 0.45	2312 ± 1.03	1.59 ± 0.51

*Average of three times ± SD.

#### Effect of solvent on rebinding

The solvent used to prepare the indacaterol standard solution can affect the strength of interaction between the polymer and the template accordingly affecting the binding capacity and selectivity of the polymer. Table S4 demonstrated that various solvents were used for the rebinding of indacaterol with the prepared polymer. The results revealed that acetonitrile exhibited the highest binding capacity and selectivity towards indacaterol among the tested solvents. This finding was because acetonitrile is a moderate polar aprotic solvent that can facilitate the formation of well-defined binding sites and maintain the stability of the pre-polymerization complex during the polymer preparation, resulting in stronger interactions between the template and functional monomers during the rebinding process^34^. Methanol, ethanol and water as solvents for rebinding showed lower binding capacity and selectivity of the polymer than acetonitrile. This can be attributed to the ability of these solvents to form a non-specific hydrogen bonding with the polymer that can affect the binding capacity and selectivity between the template and the polymer. Chloroform, DMSO and di-chloromethane showed a very low binding capacity in-addition to a poor selectivity reflected in the low IF, due to their high solvation power leading to the dissolution of the polymer.

#### Binding kinetics

Binding kinetics between the polymer and the template molecule was investigated to determine the optimal time interval for rebinding. The results in Table S5 as well as Fig. S2, revealed that the maximum binding capacity and selectivity were achieved after 120 min. A shorter time interval was inadequate for completing the rebinding process, while longer time interval led to decrease the stability of the binding complex and dissociation of the template molecule from the polymer, resulting in reduced binding capacity and selectivity^38^. Moreover, the results showed a high binding capacity and selectivity after only 5 min of contact between indacaterol and the polymer. This finding suggests that rapid mass transfer occurred during the rebinding process as a result of the high porosity and surface area of the MIP allowed for efficient diffusion of the template molecules towards the binding sites.

#### Binding capacity and affinity

**Fig. 3:**
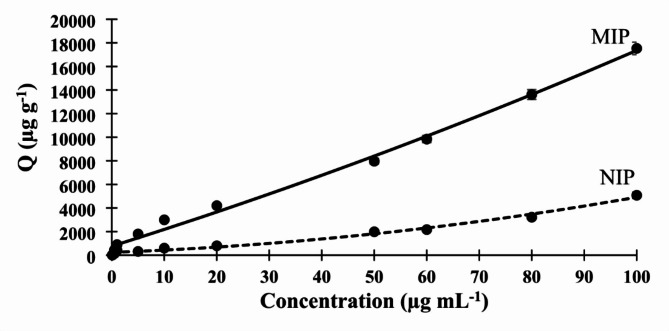
Binding isotherm of molecular imprinting and non-imprinting polymers.

The imprinting efficiency of the polymer was evaluated by analyzing its binding capacity and affinity using binding isotherm and Scatchard analysis^39^. Binding isotherm represents the correlation between the concentration of the template molecule along with the calibration curve and the binding capacity of the polymer. Figure 3 showed that as the concentration of indacaterol increased, the binding capacity of the MIP increased, which can be attributed to the high porosity and availability of binding sites in the polymer. Additionally, the overlaid plot between the MIP and its corresponding NIP demonstrated that the binding capacity of the MIP was greater than that of the NIP, indicating the high selectivity of the MIP towards indacaterol. Furthermore, the results showed that MIP had high sensitivity towards indacaterol, as it was able to bind to the template molecule at low concentrations of 0.10 µg mL^− 1^.

Scatchard analysis is a method used to determine the binding affinity, which is directly related to the association constant (K_A_) and dissociation constant (K_D_) between the template molecule and the binding site of MIP. The results of the Scatchard analysis of the MIP, Fig. S3a showed the presence of two lines representing high and low affinity binding regions. This is due to the presence of heterogeneous binding sites in the prepared polymer, which is particularly prevalent in the polymer prepared by bulk polymerization and obeys the Freundlich adsorption isotherm model. Furthermore, Table S6 provides quantitative conclusions on the values of K_D_ and Q_max_ of the MIP. To confirm that the polymer obeys heterogeneous binding model. Freundlich and Langmuir adsorption isotherms models for MIP were plotted in Fig. S3b, c and the results of both models were represented in Table S7, the value of R^2^ in the Freundlich adsorption isotherm; heterogeneous model; was found to be better than that in the Langmuir isotherm; homogenous model. Therefore, it was concluded that the MIP obeys the Freundlich adsorption isotherm model^40,41^.

#### Polymer selectivity

Table S8 demonstrated that the indacaterol polymer possesses high selectivity, as confirmed by its high binding capacity and IF towards indacaterol in the presence of co-formulated drugs in the dosage form Enerzair Breezhaler; glycopyrronium and mometasone. This selectivity is attributed to the presence of specific binding sites for indacaterol in the polymer. The same selectivity was observed in the competitive binding between indacaterol and the structurally related drug; salbutamol; which confirmed that the binding sites were selective towards indacaterol even in the presence of a structurally related drug.

The IF value of 4.53 ± 0.12 that was found during the study indicated the polymer’s significant selectivity towards indacaterol.

#### Polymer characterization

##### Fourier Transform Infrared

As shown in Fig. S4a, b. The stretching band observed at 3662 cm^− 1^ in both extracted MIP and NIP correspond to the –OH stretching of MAA, while the sharp peak at 1726 cm^− 1^ represents the –C = O stretching of EDMA. The band observed at 2988 cm^− 1^ corresponds to the stretching band of C-H due to the methyl and methylene groups present in the polymer structure. These results suggest that the FTIR spectra of extracted MIP and NIP are similar, indicating that they have the same backbone structure. This similarity serves as evidence of complete washing of the template molecule from the MIP during the washing process.

Furthermore, in Fig. S4c, d the -OH stretching band observed in indacaterol at 3662 cm^− 1^ was not present in the non-extracted MIP due to the formation of a hydrogen bond between indacaterol and MAA. Similarly, the -NH stretching band at 3332 cm^− 1^, which appeared as a sharp band in the indacaterol spectrum, was converted into a broad band in the non-extracted MIP. These findings provide evidence of the successful imprinting of the template molecule in the MIP and the formation of specific binding sites for indacaterol.

##### Scanning electron microscopy

**Fig. 4:**
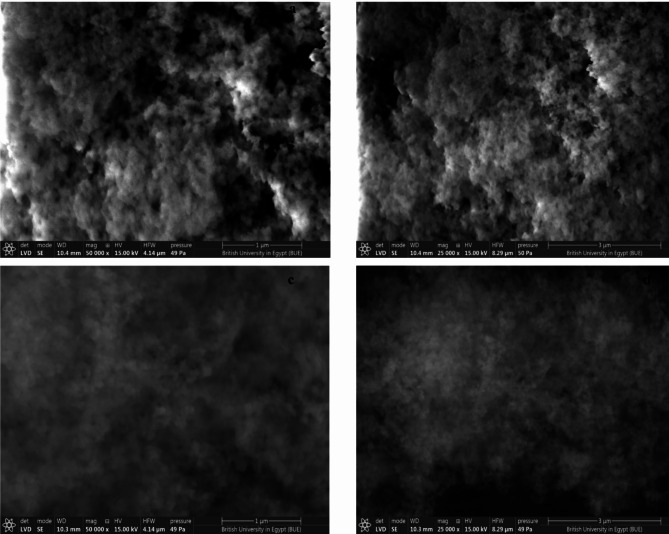
Scanning electron microscope images for molecular imprinting polymer (**a**) magnification power of 50,000 X, (**b**) magnification power of 25,000 X and non-imprinted polymer, (**c**) magnification power of 50,000X and (**d**) magnification power of 25,000X.

MIP and NIP were analyzed using SEM. The SEM characterization revealed that the surface of MIP is rough, porous, and irregular, whereas the surface of NIP is smoother and less porous. These results were shown in Fig. 4 and indicated that the template was successfully imprinted in the MIP, resulting in the formation of pores that can capture the analyte.

### Preparation and optimization of MISPE

#### Polymer weight

In this study, different weights of the polymer were investigated to determine the optimal weight that would result in the highest binding selectivity and capacity. Fig. S5 displays the results, which indicate that as the weight of the polymer increased, the binding capacity and selectivity also increased until a weight of 20.0 mg was reached. However, when using higher weights of 25.0 and 30.0 mg, the binding selectivity and capacity of the polymer decreased due to the dominance of non-specific binding sites. It is noteworthy that weights exceeding 30.0 mg resulted in cartridge blockage, which prevented the passage of solvents.

#### Cartridge capacity

In this study, the cartridge capacity was evaluated as a measure of concentration range that can be loaded into the cartridge with high selectivity. As presented in Fig. S6, the polymer exhibited a high binding capacity over a wide range of concentrations. Furthermore, high binding selectivity and capacity of the polymer was observed towards low concentrations of indacaterol, with a sensitivity reaching up to 0.10 µg mL^− 1^. The concentration of 10.0 µg mL^− 1^ of the indacaterol standard exhibited the best binding selectivity and capacity. However, increasing the concentration beyond 100.0 µg mL^− 1^ resulted in a decrease in binding capacity and selectivity, since it exceeded the capacity of the polymer’s binding sites.

#### Loading solvent

The nature of the loading solvent can influence the binding capacity and selectivity of the polymer by affecting the interaction between the analyte and the polymer in MISPE. To investigate this effect, different loading solvents were evaluated, and the results were presented in Fig. S7. The findings indicated that the best binding capacity along with the highest selectivity was achieved using acetonitrile as the loading solvent. In contrast, methanol and phosphate buffers at different pH ranges covering the acidic, neutral and basic ranges which were 3.0, 5.0, and 8.0 exhibited high binding capacity to MIP but low selectivity due to their ability to form non-specific hydrogen bonds with NIP also, at different pH values, the protonation state of the functional groups in the polymer and the analyte can also change, leading to variations in the selectivity^42^. Solvents such as DMSO, DCM, and chloroform were not used as loading solvents in this study due to their potential to dissolve the polymer and damage the binding sites.

#### Washing solvent

In this study, the washing solvent’s role in MISPE was to remove impurities present in the analyte matrix from the cartridge without interfering with the analyte binding. The washing solvent should also eliminate the analyte from the non-specific binding sites of NIP. The results presented in Fig. S8 demonstrated that water had no effect on the binding of indacaterol to MIP, but its elution power was low, rendering it incapable of washing indacaterol from non-specific binding sites of NIP.

Using methanol: water at ratios of (50: 50 and 80: 20; *by volume*), as well as acetonitrile: water at the same ratios as a washing solvent, showed the ability to wash indacaterol from non-specific binding site in NIP but also, these solvents adversely affected the binding of indacaterol in MIP due to the high solubility of indacaterol in methanol and the high elution power of acetonitrile. Conversely, using methanol: water (20: 80; *by volume*) and acetonitrile: water with the same ratio as washing solvents demonstrated a lower impact on indacaterol in MIP while washing indacaterol from NIP effectively. Acetonitrile was found to provide better results than methanol due to its lower dissolving effect on indacaterol. Additionally, the presence of the organic solvent with water was crucial to remove any organic and inorganic impurities. Accordingly, the best washing solvent was acetonitrile: water (20: 80; *by volume*).

#### Elution solvent

In this study, different solvents were evaluated for their ability to elute the template from the binding cavities of the polymer by breaking the bond between the template and the polymer as represented in Fig. S9. Methanol with varying ratios of acetic acid was used, and it was found that 1.0 mL of each solvent completely eluted the template, with no difference observed between the different ratios of acetic acid. The least ratio of acetic acid was preferred to avoid damaging the binding sites of the polymer. Additionally, the use of acetonitrile was investigated with the least ratio of acetic acid, and the percentage of eluent was found to be similar to that of methanol, but methanol was deemed to be more cost-effective.

To investigate the effect of basic pH on the elution, a basic medium using methanol: ammonia (90: 10; *by volume*) was evaluated. However, the addition of ammonia did not change the results. Therefore, it was concluded that methanol: acetic acid (90: 10; *by volume*) was the best eluting solvent.

Finally, the optimized conditions for the MISPE procedures were determined to be 20.0 mg of MIP and NIP, acetonitrile as the loading solvent, acetonitrile: water (20: 80; *by volume*) as the washing solvent, and methanol: acetic acid (90: 10; *by volume*) as the eluting solvent.

Additionally, to validate the benefit of the tailored MISPE cartridges in the selective purification of indacaterol from lung tissue samples without affecting its concentration, a lung homogenate was prepared using the same procedures as described under section "Preparation of quality control samples and lung tissue samples". However, instead of using MISPE technique, a protein precipitation technique was employed. Specifically, 930.0 µL of the lung homogenate was spiked with 60.0 µL from 1.0 mg mL^− 1^ indacaterol standard and 10.0 µL from 1.0 mg mL^− 1^ salbutamol as IS then, 2.0 mL of acetonitrile was added to the lung homogenate to precipitate any proteins, followed by centrifugation at 3000 rpm for 10.0 min. The supernatant was collected, and the solvent was evaporated to dryness. The residue was then reconstituted in 1.0 mL of methanol and vortexed for 5.0 min before injection into UPLC.

As anticipated, the resulting chromatogram was highly noisy and contained numerous impurities, Fig. 2c. Indacaterol was not detected among the noise using the protein precipitation technique, in contrast to the results obtained using the MISPE procedures. Also, the blank lung chromatogram in Fig. 2d using MISPE procedures showed no interference from any substance. These findings highlight the superiority of the MISPE technique over the protein precipitation method for the determination of indacaterol concentrations in lung tissue samples.

#### Cartridge selectivity

The selectivity of MISPE was evaluated through competitive binding between indacaterol and co-formulated drugs towards indacaterol polymer. The results demonstrated a higher loading percentage of indacaterol compared to glycopyrronium and mometasone, as the binding sites were specifically tailored for indacaterol. In the washing step, the washing solvent efficiently removed glycopyrronium and mometasone from both MIP and NIP, while only washing indacaterol from NIP without affecting its binding in MIP. This resulted in a higher percentage of eluted indacaterol compared to glycopyrronium and mometasone, as illustrated in Fig. S10. Furthermore, in competitive binding between indacaterol and salbutamol; a structurally related drug; the results showed a higher loading percentage of indacaterol compared to salbutamol. The washing solvent was able to remove salbutamol from both MIP and NIP, while only washing indacaterol from NIP, resulting in a higher percentage of eluted indacaterol relative to salbutamol as represented in Fig. S11.

IF is a parameter that indicates the selectivity of the polymer and MIP exhibited high IF during the whole study. These findings demonstrate the ability of MISPE to selectively bind and separate target analytes even in the presence of structurally related compounds.

#### Cartridge reusability

The reusability of MISPE cartridges is one of their key advantages over traditional silica-based cartridges. In this study, it was observed that each cartridge could be used up to 50 times with indacaterol standard without any reduction in the % R. Furthermore, during the bioanalytical validation using indacaterol spiked into lung tissue samples and in the pharmacokinetic study, it was observed that each cartridge could be used up to 6 times without any decrease in the % R. The cartridge can be regenerated by using 5.0 mL of methanol.

### Pharmacokinetic study of indacaterol in rat lung tissues

The lung tissue matrix presents a challenge in extracting drugs due to its complex composition and presence of various interfering components. Specifically, the presence of proteins which can bind to the drug and hinder its recovery, lipids that can cause adsorption and retention of the drug, mucus secretions, which can create a physical barrier that limits access to the drug molecules and prevents their efficient extraction, cellular components including epithelial cells, macrophages, and fibroblasts that may metabolize or sequester the drug, making it very challenging to selectively extract the target drug from the tissue matrix and extracellular matrix components such as collagen, elastin, and glycosaminoglycans that can interact with the drug, leading to binding or entrapment, and subsequently affect the extraction efficiency^43^.

To overcome this obstacle, a tailored MIP was developed specifically for indacaterol and was incorporated into MISPE, enabling the selective extraction and purification of indacaterol from the lung tissue matrix following oral inhalation. Importantly, this process does not compromise the drug’s concentration at its intended site of action.

By employing this approach, it becomes possible to comprehensively study the pharmacokinetics of indacaterol at its specific site of action within the lungs rather than relying on the drug’s concentration in the bloodstream, which could not be directly related to the desired therapeutic impact, the efficiency of the treatment depends on the drug’s concentration in the local tissue. Additionally, this methodology facilitates the determination of indacaterol’s lung deposition subsequent to oral inhalation. It has been proposed that the selective accumulation of indacaterol in lung tissue results in consistent concentrations at the desired site of action, with a delayed re-distribution through the circulation. Also, enabling the calculation of various pharmacokinetic parameters. Consequently, this provides a valuable insight into the behavior of indacaterol within the lung tissue matrix, its primary site of action and contributes to a deeper understanding of the drug’s efficacy and therapeutic implications.

**Fig. 5:**
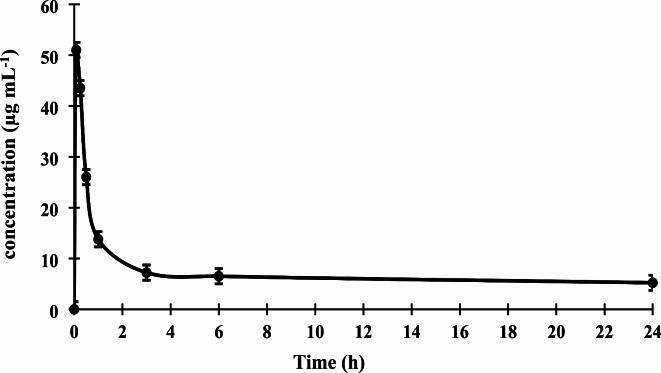
Lung concentration-time curve of indacaterol in rats following inhalation of Onbrez Breezhaler capsules contain 150 µg indacaterol.

The mean concentration-time curve of indacaterol in the rat lungs was determined after the inhalation of a single dose of Onbrez Breezhaler capsule containing 150 µg of indacaterol as represented in Fig. 5. The pharmacokinetic parameters of indacaterol were calculated and presented in Table 2. The C_max_ of indacaterol in rat lungs was (51.020 ± 2.810 µg mL^− 1^), indicating that more than 34 ± 1.880% of the nominal dose was deposited in the lung tissues. This result is consistent with a previous report that estimated the fraction of the nominal dose of inhaled indacaterol deposited and absorbed in the lungs to be approximately 34%, based on an absolute bioavailability of indacaterol of 45%^7^. The T_max_ of (0.083 ± 0.001 h) suggested that indacaterol reached its maximum concentration in the lung tissue quickly after administration, allowing for a rapid onset of therapeutic effects. The rapid absorption may be attributed to the high lung tissue penetration and lipophilicity of indacaterol.

**Table 2:** Pharmacokinetic parameters of indacaterol in rat lungs.

Pharmacokinetic parameters	Indacaterol (mean ± SD)
C_max_ (µg mL^− 1^)	51.020 ± 2.810
T_max_ (h)	0.083 ± 0.001
Elimination rate constant (h^− 1^)	0.014 ± 0.00012
t_1/2_ (h) (0–24)	48.510 ± 0.012
AUC _0−t_ (µg h mL^− 1^)	175.920 ± 1.053
AUC _0−inf_ (µg h mL^− 1^)	542.000 ± 5.245

All results were obtained by using male wistar rats (*n* = 3 per sampling time point).

The AUC _0−24_ of 175.920 ± 1.053 µg h mL^− 1^ represents the total exposure of the drug in the lungs over a period of 24 h, suggesting that indacaterol has good lung tissue penetration, rapid distribution to the site of action, and remained in the lungs for a significant period of time, which may allow for sustained therapeutic effect. The high AUC _0−inf_ of 542.000 ± 5.245 µg h mL^− 1^ suggested that indacaterol was eliminated slowly from the lung tissue, potentially allowing a prolonged therapeutic effect. The slow elimination of indacaterol may be attributed to its low clearance and high tissue binding, which may limit its availability for elimination. Clinical data indicated that uridine diphosphate glucuronyltransferase 1A1 (UGT1A1) and cytochrome P450 3A4 (CYP3A4) are the major enzymes responsible for metabolic clearance of indacaterol, studies conducted on human lung and pulmonary microsomes did not detect any apparent metabolism of indacaterol by these enzymes in the lung^7^. Therefore, it appeared that indacaterol is not locally metabolized in the lung tissue. This data is consistent with the elimination rate constant of 0.014 ± 0.00012 h^− 1^ in the current study, indicating that indacaterol is eliminated at a low rate from the lung tissue, potentially allowing for sustained therapeutic effects. The half-life of 48.510 ± 0.012 h also suggests that indacaterol is eliminated from the lung tissue at a low rate.

These pharmacokinetic parameters suggest that indacaterol has good lung tissue penetration, is rapidly distributed to the site of action, and may allow for sustained therapeutic effects while minimizing the risk of accumulation and potential side effects. When β_2_ agonists are administered by inhalation, the desired therapeutic effects on airways depend on local tissue concentrations, which may not be directly related to blood drug concentrations so, the accurate measurement of drug concentration in its site of action is critically needed.

## Conclusion

A molecular imprinted polymer was synthesized using indacaterol as a template. This polymer was characterized by high binding capacity and selectivity toward indacaterol. The synthesized polymer was used as a sorbent in SPE. MISPE procedures were optimized for selective extraction and purification of indacaterol from rat lungs using 20.0 mg of MIP and NIP, acetonitrile as the loading solvent, acetonitrile: water (20: 80; *by volume*) as the washing solvent, and methanol: acetic acid (90: 10; *by volume*) as the eluting solvent. A pharmacokinetic study was then performed to determine the mean concentration-time curve of indacaterol in the rat lungs and to determine the lung deposition of indacaterol after the inhalation of a single dose of Onbrez Breezhaler capsule containing 150 µg of the drug. It was observed that about 34% of indacaterol was deposited in lung tissues with rapid onset which is 5 min. The pharmacokinetic parameters suggested that indacaterol has good lung tissue penetration, rapid distribution to the site of action, and sustained therapeutic effects with minimum risk of accumulation and potential side effects. A developed and bioanalytically validated UPLC method was used for the analysis of indacaterol during the whole study.

## Electronic supplementary material

Below is the link to the electronic supplementary material.

Supplementary Material 1

## Data Availability

Data will be made available on request. The request for materials should be addressed to M.T. (mohamed.tarek@bue.edu.eg)

## References

[1] National center for biotechnology information, PubChem compound summary for CID 6918554, Indacaterol. (2024). https://pubchem.ncbi.nlm.nih.gov/compound/Indacaterol.

[2] Battram, C. *et al.* In vitro and in vivo pharmacological characterization of 5-[(R)-2-(5, 6-diethyl-indan-2-ylamino)-1-hydroxy-ethyl]-8-hydroxy-1H-quinolin-2-one (indacaterol), a novel inhaled β2 adrenoreceptor agonist with a 24-h duration of action. *J. Pharmacol.* **317**, 762–770. 10.1124/jpet.105.098251 (2006). 10.1124/jpet.105.098251 16434564

[3] Kagan, M., Dain, J., Peng, L. & Reynolds, C. Metabolism and pharmacokinetics of indacaterol in humans. *Drug Metab. Dispos.* **40**, 1712–1722. 10.1124/dmd.112.046151 (2012). 22648561 10.1124/dmd.112.046151

[4] Johnson, M. Beta_2_-adrenoceptors: Mechanisms of action of beta_2_-agonists. *Paediatr.* **2**, 57–62. 10.1053/prrv.2000.0102 (2001). 10.1053/prrv.2000.0102 16263481

[5] Cazzola, M. & Matera, M. G. Novel long-acting bronchodilators for COPD and asthma. *Br J Pharmacol.* **155**, 291–299. 10.1038/bjp.2008.284 (2008). 18604231 10.1038/bjp.2008.284 PMC2567883

[6] Tamura, G. & Ohta, K. Adherence to treatment by patients with asthma or COPD: Comparison between inhaled drugs and transdermal patch. *Respir Med.* **101**, 1895–1902. 10.1016/j.rmed.2007.05.001 (2007). 17587559 10.1016/j.rmed.2007.05.001

[7] Cazzola, M., Calzetta, L., Page, C. P. & Matera, M. G. Use of indacaterol for the treatment of COPD: A pharmacokinetic evaluation. *Expert Opin Drug Metab. Toxicol* **10**, 129–137. 10.1517/17425255.2014.865723 (2014). 24295085 10.1517/17425255.2014.865723

[8] Lombardi, D., Cuenoud, B. & Kramer, S. D. Lipid membrane interactions of indacaterol and salmeterol: Do they influence their pharmacological properties?. *Eur J Pharm Sci.* **38**, 533–547. 10.1016/j.ejps.2009.10.001 (2009). 19819331 10.1016/j.ejps.2009.10.001

[9] Lasáková, M. & Jandera, P. Molecularly imprinted polymers and their application in solid phase extraction. *J. Sep. Sci.* **32**, 799–812. 10.1002/jssc.200800506 (2009). 19219838 10.1002/jssc.200800506

[10] Holthoff, E. L. & Bright, F. V. Molecularly templated materials in chemical sensing. *Anal. Chim. Acta.* **594**, 147–161. 10.1016/j.aca.2007.05.044 (2007). 17586109 10.1016/j.aca.2007.05.044

[11] Li, Y. *et al.* Synthesis of metal-organic framework@ molecularly imprinted polymer adsorbents for solid phase extraction of organophosphorus pesticides from agricultural products. *J. Chromatogr. B.*10.1016/j.jchromb.2021.123081 (2022). 10.1016/j.jchromb.2021.123081 34911000

[12] Badawy, M. E., El-Nouby, M. A., Kimani, P. K., Lim, L. W. & Rabea, E. I. A review of the modern principles and applications of solid-phase extraction techniques in chromatographic analysis. *Anal Sci.* **38**, 1457–1487. 10.1007/s44211-022-00190-8 (2022). 36198988 10.1007/s44211-022-00190-8 PMC9659506

[13] Gaho, M. M. *et al.* Synthesis of novel magnetic molecularly imprinted polymers by solid-phase extraction method for removal of norfloxacin. *Chin. J. Anal. Chem.*10.1016/j.cjac.2022.100079 (2022).

[14] Kakkar, V. & Narula, P. Role of molecularly imprinted hydrogels in drug delivery-A current perspective. *Int. J. Pharm.*10.1016/j.ijpharm.2022.121883 (2022). 35870667 10.1016/j.ijpharm.2022.121883

[15] Gupta, B. D. & Semwal, V. Recent advances in molecular imprinting technique based fiber optic biosensors. *Opt. Fiber Technol.* **80**, 555. 10.1016/j.yofte.2023.103429 (2023).

[16] Akgönüllü, S., Özgür, E. & Denizli, A. Recent advances in quartz crystal microbalance biosensors based on the molecular imprinting technique for disease-related biomarkers. *Chemosensors.* **10**, 106–116. 10.3390/chemosensors10030106 (2022).

[17] Ҫimen, D., Bereli, N., Günaydın, S. & Denizli, A. Molecular imprinted nanoparticle assisted surface plasmon resonance biosensors for detection of thrombin. *Talanta.*10.1016/j.talanta.2022.123484 (2022). 35462248 10.1016/j.talanta.2022.123484

[18] Ghany, M. F., Hussein, L. A., Magdy, N. & Yamani, H. Z. Simultaneous spectrophotometric determination of indacaterol and glycopyrronium in a newly approved pharmaceutical formulation using different signal processing techniques of ratio spectra. *Spectrochim. Acta A.* **157**, 251–257. 10.1016/j.saa.2016.01.002 (2016). 10.1016/j.saa.2016.01.002 26779820

[19] El-Ashry, S. M., El-Wasseef, D. R., El-Sherbiny, D. T. & Salem, Y. A. Spectrophotometric and spectrofluorimetric determination of indacaterol maleate in pure form and pharmaceutical preparations: Application to content uniformity. *Lumin.* **30**, 891–897. 10.1002/bio.2838 (2015). 10.1002/bio.2838 25620654

[20] El-Abasawy, N. M., Attia, K. A., Abouserie, A. A., El-Olemy, A. & Elsayed, A. O. RP-HPLC-DAD method for the simultaneous quantification of indacaterol and glycopyrronium in their pharmaceutical formulation. *WJPPS.* **7**, 166–176. 10.20959/wjpps20183-11151 (2018).

[21] Salem, Y. A., El-Sherbiny, D. T., El-Wasseef, D. R. & El-Ashry, S. M. HPLC determination of indacaterol maleate in pharmaceutical preparations adopting ultraviolet and fluorescence detection. *Int J Pharm Sci Res.* **6**, 1324–1332 (2015).

[22] Ammari, W. G. *et al.* Indacaterol determination in human urine: validation of a liquid–liquid extraction and liquid chromatography-tandem mass spectrometry analytical method. *J. Aerosol Med. Pulm. Drug Deliv.* **28**, 202–210. 10.1089/jamp.2014.1153 (2015). 25229261 10.1089/jamp.2014.1153

[23] Bartels, C., Jain, M., Yu, J., Tillmann, H. C. & Vaidya, S. Population pharmacokinetic analysis of indacaterol/glycopyrronium/mometasone furoate after administration of combination therapies using the breezhaler device in patients with asthma. *Eur. J. Drug Metab. Pharmacokinet.* **46**, 487–504. 10.1007/s13318-021-00689-x (2021). 34024035 10.1007/s13318-021-00689-x PMC8298373

[24] Vaidya, S. *et al.* Pharmacokinetics of indacaterol, glycopyrronium and mometasone furoate following once-daily inhalation as a combination in healthy subjects. *Pulm. Pharmacol. Ther.*10.1016/j.pupt.2020.101964 (2020). 33035700 10.1016/j.pupt.2020.101964

[25] Vutukuri, N. & Ajitha, M. Stability indicating RP-HPLC method development and validation for simultaneous estimation of indacaterol and glycopyrrolate and in bulk and pharmaceutical dosage form. *WJPPS.* **10**, 74–81. 10.54037/WJPS.2022.100107 (2022).

[26] Yerra, N. V. *et al.* Identification and characterization of degradation products of indacaterol using liquid chromatography/mass spectrometry. *Eur. J. Mass Spectrom.* **26**, 425–431. 10.1177/1469066720971550 (2020). 10.1177/1469066720971550 33153322

[27] Inoue, S. *et al.* Pharmacokinetics of indacaterol, glycopyrronium and mometasone furoate administered as an inhaled fixed-dose combination in Japanese and Caucasian healthy subjects. *BMC Pulm. Med.*10.1186/s12890-020-01382-6 (2021). 33413291 10.1186/s12890-020-01382-6 PMC7791651

[28] Emotte, C. *et al.* Validation of an on-line solid-phase extraction method coupled to liquid chromatography-tandem mass spectrometry detection for the determination of Indacaterol in human serum. *J. Chromatogr. B.* **895–896**, 1–9. 10.1016/j.jchromb.2012.02.025 (2012). 10.1016/j.jchromb.2012.02.025 22483398

[29] Sobolevsky, T. & Ahrens, B. High-throughput liquid chromatography tandem mass spectrometry assay as initial testing procedure for analysis of total urinary fraction. *Drug Test. Anal.* **13**, 283–298. 10.1002/dta.2917 (2021). 32852861 10.1002/dta.2917

[30] Bozzolino, C., Leporati, M., Gani, F., Ferrero, C. & Vincenti, M. Development and validation of an UHPLC–MS/MS method for β_2_-agonists quantification in human urine and application to clinical samples. *J. Pharm. Biomed. Anal.* **150**, 15–24. 10.1016/j.jpba.2017.11.055 (2018). 29202304 10.1016/j.jpba.2017.11.055

[31] Tarek, M., Ghoniem, N. S., Hegazy, M. A. & Wagdy, H. A. Design of experiment-based green UPLC-DAD method for the simultaneous determination of indacaterol, glycopyrronium and mometasone in their combined dosage form and spiked human plasma. *JCS.*10.1093/chromsci/bmad072 (2023). 10.1093/chromsci/bmad072 37635399

[32] U.D.o. Health, H. Services. Bioanalytical method validation guidance for industry. US Department of Health and Human Services, Food and Drug Administration. *Center for Drug Evaluation and Research and Center for veterinary Medicines*. http://www.fda.gov/cder/guidance/4252fnl.htm (2001).

[33] Vasapollo, G. *et al.* Molecularly imprinted polymers: present and future prospective. *Int J Mol Sci.* **12**, 5908–5945. 10.3390/ijms12095908 (2011). 22016636 10.3390/ijms12095908 PMC3189760

[34] Meier, F. & Mizaikoff, B. Molecularly imprinted polymers as artificial receptors. *Artif. Receptors Chem. Sens.*10.1002/9783527632480.ch13 (2010).

[35] Zhang, Q. *et al.* Amino terminated supramolecular cucurbit [6] uril pseudorotaxane complexes immobilized on magnetite@ silica nanoparticles: A highly efficient sorbent for salvianolic acids. *Talanta.* **195**, 354–365. 10.1016/j.talanta.2018.11.086 (2019). 30625555 10.1016/j.talanta.2018.11.086

[36] Bae, J. W., Kim, M. J., Jang, C. G. & Lee, S. Y. Determination of meloxicam in human plasma using a HPLC method with UV detection and its application to a pharmacokinetic study. *J. Chromatogr. B.* **859**, 69–73. 10.1016/j.jchromb.2007.09.004 (2007). 10.1016/j.jchromb.2007.09.004 17921074

[37] Yilmaz, E., Mosbach, K. & Haupt, K. Influence of functional and crosslinking monomers and the amount of template on the performance of molecularly imprinted polymers in binding assays. *Anal. Commun.* **36**, 167–170 (2019).

[38] Tarek, M., Elzanfaly, E. S., Amer, S. M. & Wagdy, H. A. Selective analysis of Nadifloxacin in Human plasma samples using a molecularly imprinted polymer-based solid-phase extraction proceeded by UPLC-DAD analysis. *Microchem. J.*10.1016/j.microc.2020.105162 (2020).

[39] Umpleby, R. J. *et al.* Recognition directed site-selective chemical modification of molecularly imprinted polymers. *Macromol.* **34**, 8446–8452. 10.1021/ma010903s (2001).

[40] Matsui, J., Miyoshi, Y., Doblhoff-Dier, O. & Takeuchi, T. A molecularly imprinted synthetic polymer receptor selective for atrazine. *Anal. Chem.* **67**, 4404–4408. 10.1021/ac00119a032 (1995).

[41] Umpleby, R. J. *et al.* Characterization of the heterogeneous binding site affinity distributions in molecularly imprinted polymers. *J Chromatogr B.* **804**, 141–149. 10.1016/j.jchromb.2004.01.064 (2004). 10.1016/j.jchromb.2004.01.064 15093168

[42] Wu, L., Zhu, K., Zhao, M. & Li, Y. Theoretical and experimental study of nicotinamide molecularly imprinted polymers with different porogens. *Anal Chim Acta.* **549**, 39–44. 10.1016/j.aca.2005.06.009 (2005).

[43] Knudsen, L. & Ochs, M. The micromechanics of lung alveoli: Structure and function of surfactant and tissue components. *Histochem. Cell Biol.* **150**, 661–676. 10.1007/s00418-018-1747-9 (2018). 30390118 10.1007/s00418-018-1747-9 PMC6267411

