# Supplementary material for: Pharmacokinetics of pulmonary indacaterol in rat lung using molecular imprinting solid-phase extraction coupled with RP-UPLC
Source: Sci Rep. 2024 Oct 4;14:23126. doi: 10.1038/s41598-024-72822-0 (PMC11452728; doi:10.1038/s41598-024-72822-0)
Supplement: Supplementary file 1 — Supplementary Material 1 [file 41598_2024_72822_MOESM1_ESM.docx]

**Supplementary file**

**Pharmacokinetics of pulmonary indacaterol in rat lung using molecular imprinting solid-phase extraction coupled with RP-UPLC**

Mohamed Tarek^1, 2^, Nermine S. Ghoniem^3*^, Maha A. Hegazy^4^, Hebatallah A. Wagdy^1, 2**^

^1^Pharmaceutical Chemistry Department, Faculty of Pharmacy, The British University in Egypt (BUE), Cairo, Egypt.

^2^Health Research Center of Excellence; Drug Research and Development Group, Faculty of Pharmacy, The British University in Egypt, Cairo, Egypt.

^3^Analytical Chemistry Department, Faculty of Pharmacy, Cairo University, Kasr-El Aini Street, 11562 Cairo, Egypt.

^4^Pharmaceutical Chemistry Department, Faculty of Pharmacy, Future University in Egypt, Cairo 11835, Egypt.

*** Corresponding Author**

Nermine S. Ghoniem

Department of Analytical Chemistry, Faculty of Pharmacy, Cairo University.

E-mail address: [nermine.ghoniem@pharma.cu.edu.eg](mailto:nermine.ghoniem@pharma.cu.edu.eg)

^**^ **Corresponding Author**

Hebatallah A. Wagdy

Department of Pharmaceutical Chemistry, Faculty of Pharmacy, The British University in Egypt.

E-mail address: [Hebatallah.wagdy@bue.edu.eg](mailto:Hebatallah.wagdy@bue.edu.eg)

**List of tables**

**Table S1:** Intra-day and inter-day accuracy and precision of the LLOQ and QCs of indacaterol

**Table S2:** Stability of the lung samples spiked with indacaterol standard under different conditions.

**Table S3:** Extraction recovery of indacaterol from lung samples.

**Table S4:** Effect of solvent on the rebinding of indacaterol to the polymer.

**Table S5:** Binding kinetics of indacaterol polymer.

**Table S6:** K_D_ and Q_max_, for lower and higher affinity binding sites of the MIP at equilibrium conditions.

**Table S7:** Adsorption isotherms parameters for MIP.

**Table S8:** Binding selectivity of the prepared polymer.

**List of figures**

**Fig. S1.:** Calibration curve of indacaterol in lung tissue sample in the concentrations ranging from 0.10 to 100.0 µg mL^-1^ using salbutamol as IS**.**

**Fig. S2:** Binding kinetics of indacaterol polymer.

**Fig. S3:** (**a**) Scatchard plot and (**b**) Freundlich and (**c**) Langmuir adsorption isotherms models for molecular imprinting polymer.

**Fig. S4:** Fourier transform infrared spectra of (**a**) extracted molecular imprinting polymer, (**b**) non-imprinted polymer, (**c**) indacaterol and (**d**) non-extracted molecular imprinting polymer.

**Fig. S5:** Effect of polymer weight on the loading % of indacaterol.

**Fig. S6:** Effect of different concentrations of indacaterol on the % loaded to molecular imprinting solid-phase extraction cartridge.

**Fig. S7:** Effect of type of loading solvent on the % loaded of indacaterol to molecular imprinting solid-phase extraction cartridge.

**Fig. S8:** Effect of type of washing solvent on the % washed of indacaterol from molecular imprinting solid-phase extraction cartridge.

**Fig. S9:** Effect of type of eluting solvent on the % eluted of indacaterol from molecular imprinting solid-phase extraction cartridge.

**Fig. S10:** Recovery of (**a**) indacaterol, glycopyrronium and mometasone mixture of concentration 40.0 µg mL^-1^ from molecular imprinting polymer and non-imprinting polymer after loading, (**b**) washing with 1.0 mL acetonitrile: water (20 :80; *by volume*) and (**c**) elution with 1.0 mL methanol: acetic acid (90: 10; *by volume*) using 20.0 mg of indacaterol polymer.

**Fig. S11:** Recovery of (**a**) indacaterol and salbutamol mixture of concentration 40.0 µg mL^-1^ from molecular imprinting polymer and non-imprinting polymer after loading, (**b**) washing with 1.0 mL acetonitrile: water (20 :80; *by volume*) and (**c**) elution with 1.0 mL methanol: acetic acid (90: 10; *by volume*) using 20.0 mg of indacaterol polymer.

| **Table S1:** Intra-day and inter-day accuracy and precision of the LLOQ and QCs of indacaterol | | | | | |
| --- | --- | --- | --- | --- | --- |
|  | **Concentration**  **(µg mL^-1^)** | **Intra-day accuracy**  **% R*** | **Inter-day accuracy**  **% R*** | **Intra-day precision**  **% RSD*** | **Inter-day precision**  **% RSD*** |
| **LLOQ** | 0.10 | 112.00 | 113.05 | 4.34 | 5.02 |
| **QCL** | 0.30 | 106.00 | 107.12 | 2.14 | 3.53 |
| **QCM** | 35.00 | 104.58 | 105.32 | 2.04 | 3.21 |
| **QCH** | 70.00 | 101.36 | 102.17 | 1.95 | 2.19 |
| *Average of 6 determinations | | | | | |

| **Table S2:** Stability of the lung samples spiked with indacaterol standard under different conditions | |
| --- | --- |
| Item | % deviation from fresh sample |
| **Short-term stability** | |
| LLOQ | -11.30±3.50 |
| QCL | -5.74±2.25 |
| QCM | -4.14±2.61 |
| QCH | -2.89±1.94 |
| **Auto-sampler stability** | |
| LLOQ | -12.05±4.20 |
| QCL | -5.98±2.58 |
| QCM | -4.02±2.34 |
| QCH | -1.71±2.67 |
| **Freeze and thaw stability** | |
| LLOQ | -13.26±4.85 |
| QCL | -4.80±3.26 |
| QCM | -4.30±2.81 |
| QCH | -2.20±2.25 |
| **Long-term stability** | |
| LLOQ | -13.10±5.89 |
| QCL | -4.40±3.88 |
| QCM | -3.90±2.22 |
| QCH | -3.28±1.32 |
| % Deviation: (% R of stability samples - % R of fresh samples/ % R of fresh samples) × 100. | |

| **Table S3:** Extraction recovery of indacaterol from lung samples | | |
| --- | --- | --- |
|  | **Concentration (µg mL^-1^)** | **% R±SD**^*^ |
| **LLOQ** | 0.10 | 111.58**±**4.98 |
| **QCL** | 0.30 | 105.28**±**2.54 |
| **QCM** | 35.00 | 103.73**±**2.08 |
| **QCH** | 70.00 | 100.71**±**1.86 |
| *Average of 6 determinations | | |

| **Table S4:** Effect of solvent on the rebinding of indacaterol to the polymer | | | |
| --- | --- | --- | --- |
| **Type of solvent** | **Q_MIP_ (µg g^-1^)^*^** | **Q_NIP_ (µg g^-1^)^*^** | **IF^*^** |
| **Acetonitrile** | 9840±0.86 | 2172±0.74 | 4.53±0.12 |
| **Methanol** | 3285±0.42 | 1314±0.36 | 2.50±0.06 |
| **Ethanol** | 3420±0.67 | 1352±0.33 | 2.53±0.34 |
| **Water** | 928±0.21 | 890±0.60 | 1.04±0.39 |
| **Chloroform** | 22±0.32 | 23±0.22 | 0.96±0.10 |
| **Di-chloromethane** | 12±0.36 | 13.10±0.47 | 0.92±0.11 |
| **Dimethyl sulfoxide** | 8±0.54 | 8.52±0.66 | 0.94±0.12 |
| *****Average of three times ± SD | | | |

| **Table S5:** Binding kinetics of indacaterol polymer | | | |
| --- | --- | --- | --- |
| **Time (min)** | **Q_MIP_ (µg g^-1^)^*^** | **Q_NIP_ (µg g^-1^)^*^** | **IF^*^** |
| **5** | 758±0.98 | 195±0.36 | 3.89±0.62 |
| **15** | 1245±0.24 | 318±0.54 | 3.92±0.3 |
| **30** | 3014±0.47 | 752±0.72 | 4.01±0.25 |
| **60** | 5065±0.35 | 1248±0.36 | 4.06±0.01 |
| **120** | 9840±0.86 | 2172±0.74 | 4.53±0.12 |
| **240** | 6512±0.75 | 1637±0.11 | 3.98±0.64 |
| **360** | 3125±0.36 | 783±0.25 | 3.99±0.11 |
| **480** | 1542±0.21 | 398±0.36 | 3.87±0.15 |
| **1080** | 1024±0.36 | 272±0.82 | 3.76±0.46 |
| **1200** | 945±0.87 | 244±0.75 | 3.87±0.12 |
| **1440** | 942±0.21 | 240±0.47 | 3.92±0.26 |
| *Average of three times ± SD | | | |

| **Table S6:** K_D_ and Q_max_, for lower and higher affinity binding sites of the MIP at equilibrium conditions | | |
| --- | --- | --- |
|  | **Line 1: high affinity region** | **Line 2: low affinity region** |
| **Linear regression line equation** | $y=$−0.9708$x+4499.9$ | $y=0.0691x+218.39$ |
| **R^2^** | 0.9857 | 0.9825 |
| **Equilibrium dissociation constant K_D_ (μg)** | 1.03±0.12 | 14.47±0.67 |
| **Maximum binding sites Q_max_ (μg g^-1^)** | 4635±0.13 | 3160±0.32 |

| **Table S7**: Adsorption isotherms parameters for MIP. | | | | | |
| --- | --- | --- | --- | --- | --- |
| **Freundlich** | | | **Langmuir** | | |
| K_F_ (µg^1-1/n^ mL^1/n^ g^-1^) | n | R^2^ | Q_max_ (µg g^-1^) | K_L_ (mL µg ^-1^) | R^2^ |
| 171.5 | 1.1 | 0.9342 | 10000 | 0.043 | 0.8287 |

| **Table S8:** Binding selectivity of the prepared polymer | | | | | | | | | | | | | |
| --- | --- | --- | --- | --- | --- | --- | --- | --- | --- | --- | --- | --- | --- |
| **Concentration**  **(µg mL^-1^)** | **Competitive binding between indacaterol, glycopyrronium and mometasone to indacaterol polymer** | | | | | | | | | | | | |
|  | **Indacaterol** | | | | | **Glycopyrronium** | | | | **Mometasone** | | | |
|  | **Q_MIP_**  **(µg g^-1^)^*^** | **Q_NIP_**  **(µg g^-1^)^*^** | | **IF^*^** | | **Q_MIP_**  **(µg g^-1^)^*^** | **Q_NIP_**  **(µg g^-1^)^*^** | **IF^*^** | | **Q_MIP_**  **(µg g^-1^)^*^** | **Q_NIP_**  **(µg g^-1^)^*^** | | **IF^*^** |
| 40.0 | 6247±0.36 | 1671±0.66 | | 3.73±0.3 | | 45±0.21 | 43±0.61 | 1.05±0.4 | | 51±0.91 | 50±0.42 | | 1.02±0.4 |
|  | **Competitive binding between indacaterol and salbutamol to indacaterol polymer** | | | | | | | | | | | | |
|  | **Indacaterol** | | | | | | **Salbutamol** | | | | | | |
|  | **Q_MIP_**  **(µg g^-1^)^*^** | | **Q_NIP_**  **(µg g^-1^)^*^** | | **IF^*^** | | **Q_MIP_**  **(µg g^-1^)^*^** | | **Q_NIP_**  **(µg g^-1^)^*^** | | | **IF^*^** | |
|  | 6252±0.35 | | 1654±0.61 | | 3.78±0.26 | | 94±0.54 | | 92±0.68 | | | 1.02±0.14 | |
| *Average of three times ± SD | | | | | | | | | | | | | |

**Fig. S1**

NIP

MIP

**Fig. S2**

Low affinity binding region

High affinity binding region

**a**

**c**

**b**

**Fig. S3**

**
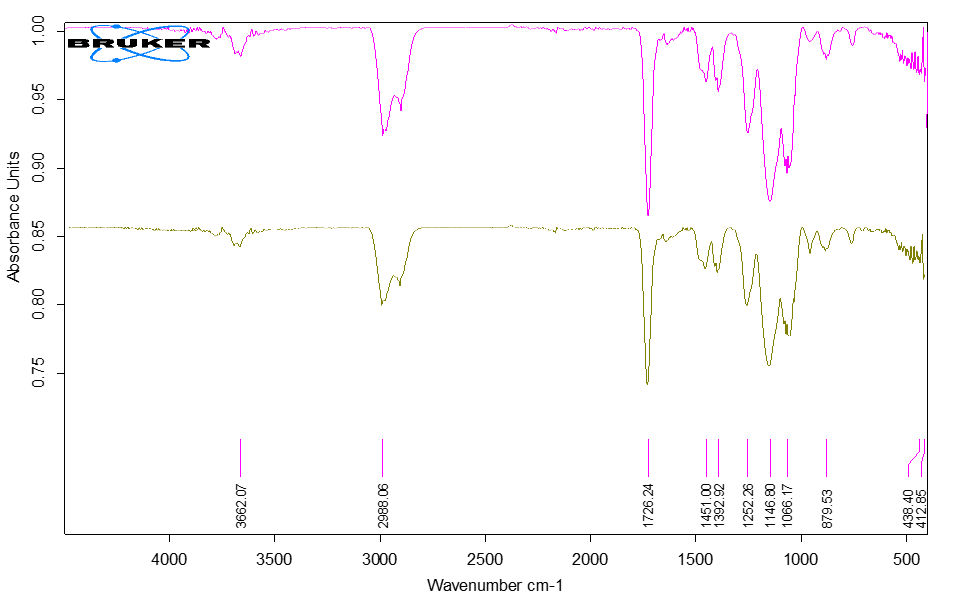

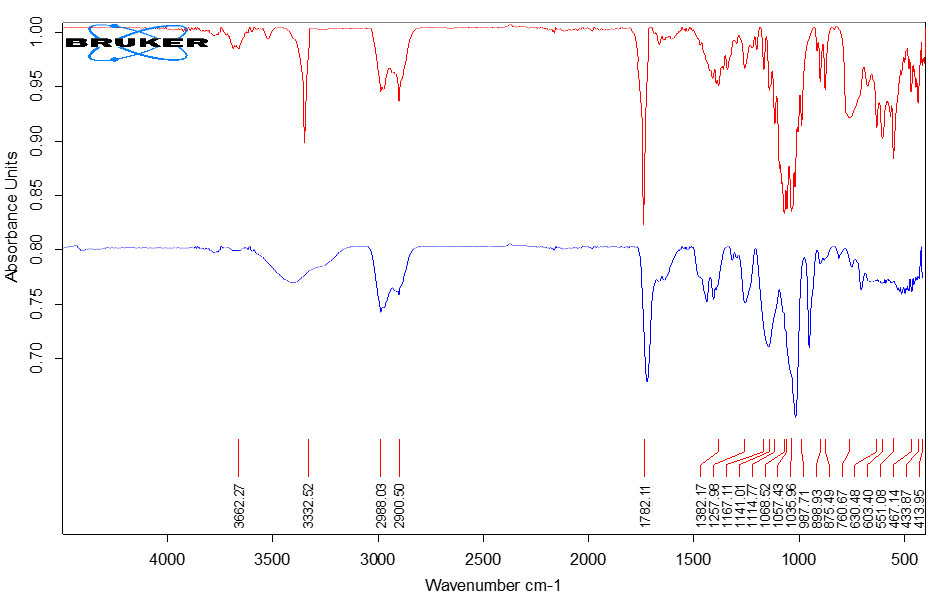
**

**c**

**a**

**b**

**d**

**Fig. S4**

**Fig. S5**

**Fig. S6**

**Fig. S7**

**Fig. S8**

**Fig. S9**

**a**

**b**

**c**

**Fig. S10**

**c**

**b**

**a**

**Fig. S11**
